# Early anhedonia distinguishes behavioral variant frontotemporal dementia from Alzheimer's disease

**DOI:** 10.1002/dad2.70501

**Published:** 2026-10-09

**Authors:** Siobhán Shaw, Samuel L Warren, Claris Teng, Jody Huynh, Rebekah Ahmed, Olivier Piguet, Alexis E Whitton, Muireann Irish

**Affiliations:** ^1^ Brain and Mind Centre The University of Sydney Sydney New South Wales Australia; ^2^ School of Psychology The University of Sydney Sydney New South Wales Australia; ^3^ School of Medical Sciences The University of Sydney Sydney New South Wales Australia; ^4^ Black Dog Institute University of New South Wales Sydney New South Wales Australia

**Keywords:** Alzheimer's disease (AD), anhedonia, behavioral variant frontotemporal dementia (bvFTD), differential diagnosis, event‐based modeling, neuropsychiatric symptoms

## Abstract

**INTRODUCTION:**

Differentiating behavioral variant frontotemporal dementia (bvFTD) from Alzheimer's disease (AD) remains challenging due to overlapping early behavioral and cognitive features. Anhedonia has emerged as a promising clinical marker, yet its multidimensional nature and temporal emergence across dementia syndromes remain unclear.

**METHODS:**

Anticipatory and consummatory components of anhedonia were assessed in bvFTD and AD using the Temporal Experience of Pleasure Scale. Event‐based modeling was applied in each group to stage the probabilistic emergence of anticipatory and consummatory anhedonia dimensions relative to apathy and cognitive symptoms.

**RESULTS:**

Relative to controls, bvFTD showed marked loss of pleasure across anticipatory and consummatory components, whereas AD showed selective anticipatory anhedonia in the context of preserved consummatory pleasure. Event‐based modeling placed consummatory and anticipatory anhedonia dimensions as early features of bvFTD occurring prior to overt cognitive changes. In contrast, for AD, anhedonia was probabilistically more likely to arise at later stages of the disease sequence, subsequent to cognitive decline.

**DISCUSSION:**

These findings highlight consummatory anhedonia as an early, clinically informative marker of bvFTD and support the value of multidimensional hedonic assessment for improving dementia differentiation.

## Highlights

- Multidimensional anhedonia differs across bvFTD and AD.
- bvFTD shows global deficits in anticipatory and consummatory pleasure.
- AD shows anticipatory deficits with preserved consummatory pleasure.
- Anticipatory and consummatory anhedonia emerge early in bvFTD and later in AD.
- EBM identifies anhedonia as a marker for early dementia differentiation

## 1 BACKGROUND

Distinguishing behavioral variant frontotemporal dementia (bvFTD) from Alzheimer's disease (AD) is a major challenge in dementia diagnosis, particularly in early stages when behavioral, neuropsychiatric, and cognitive phenotypes overlap.^1^, ^2^, ^3^ Current core diagnostic criteria for bvFTD rely on the presence of apathy, loss of empathy, executive dysfunction, and disinhibition, in the context of relatively spared episodic memory and visuospatial function, for a diagnosis of bvFTD.^4^, ^5^ Mounting evidence, however, indicates that many of these cognitive and behavioral symptoms are present in AD^6^, ^7^, ^8^ and other mid‐life neuropsychiatric disorders.^9^ As a result, individuals with bvFTD face protracted diagnostic delays or are commonly misdiagnosed with AD,^3^ limiting timely access to appropriate care and support.^10^, ^11^ Identifying novel clinical features that can better distinguish these syndromes early in the disease course represents a crucial unmet need, one that is particularly important in clinical settings where neuroimaging and biomarker information is unavailable.

The neuropsychiatric symptom of anhedonia has emerged as a potentially crucial but overlooked feature of FTD.^12^, ^13^, ^14^ Defined as a diminished capacity to experience or anticipate pleasure,^15^ anhedonia has traditionally been conceptualized as a core feature of depression and other primary psychiatric disorders.^16^, ^17^ Although depressive symptoms may co‐occur in dementia, growing evidence indicates that anhedonia and depression represent related but dissociable constructs, with anhedonia reflecting a distinct disturbance in reward processing rather than simply a manifestation of depressed mood.^12^ In dementia syndromes, however, reduced interest or disengagement from previously enjoyable activities is almost universally subsumed under the construct of “apathy,” a core diagnostic feature of bvFTD.^5^

This conflation of apathy and anhedonia obscures an important distinction. Contemporary neuropsychiatric research demonstrates that apathy and anhedonia are dissociable constructs with partially distinct behavioral and neural correlates.^18^ Apathy refers primarily to reduced initiation and engagement in goal‐directed behavior and is present from early in the disease course in both bvFTD and AD.^8^, ^12^, ^19^, ^20^ The presence of apathy, however, does not by default denote the presence of anhedonia. Empirical evidence shows that although apathy and anhedonia are present in bvFTD, these symptoms are dissociable in AD, where apathy occurs in the relative absence of anhedonia.^12^ This behavioral dissociation is further reflected on the neural level, with apathy and anhedonia associated with partially separable brain networks.^12^, ^14^ Indeed, recent studies indicate that apathy and anhedonia predict different functional outcomes across different dementia syndromes.^13^ Taken together, these findings suggest that while apathy is a common feature of neurodegenerative disorders, anhedonia likely represents a specific early behavioral signature of bvFTD.

Crucially, anhedonia is not a unitary construct but rather encompasses separable components of reward processing, including an anticipatory component (i.e., the ability to anticipate a reward/pleasurable outcome) and a consummatory phase (i.e., the in‐the‐moment experience of reward/pleasure).^21^, ^22^, ^23^ Anticipatory pleasure relies, in part, on the capacity to construct a specific, vivid, and personally meaningful representation of a future positive event and to pre‐experience its likely affective value.^24^ When future events are represented with less detail, imagery, plausibility, or personal significance, they may generate less positive affect during anticipation.^24^ In contrast, consummatory pleasure reflects the immediate sensory and affective experience of reward in the moment and may therefore be less reliant on higher‐order cognitive processes.^23^ Indeed, evidence from psychiatric populations suggests these components can be dissociable, with impairments manifesting in one domain (typically anticipatory pleasure) in the absence of impairments in the other (i.e., consummatory pleasure).^25^, ^26^

Adopting a multidimensional framework is particularly pertinent to bvFTD, where early neurodegeneration targets orbitofrontal and frontostriatal circuits central to reward valuation and anticipation.^19^, ^27^, ^28^, ^29^ In contrast, these reward networks appear to be relatively preserved in the early stages of AD.^28^, ^29^ Despite clear differences, prior studies treated anhedonia as a single construct, potentially obscuring important syndrome‐specific patterns of impairment. Such distinctions may be particularly valuable for refining clinical phenotypes and improving diagnostic precision in bvFTD.

The objectives of this study were two‐fold. First, we examined profiles of anticipatory and consummatory anhedonia in AD and bvFTD using the Temporal Experience of Pleasure Scale (TEPS).^23^ Second, we leveraged event‐based modeling (EBM) as a data‐driven approach to estimate the probabilistic ordering of clinical features, including anhedonia in each dementia subtype. This method enabled us to stage the probabilistic emergence of clinical symptoms relative to one another without requiring longitudinal data. We hypothesized that bvFTD would exhibit broad impairments across both anticipatory and consummatory dimensions of pleasure, reflecting previously reported disturbances in future‐event simulation and reward processing in this syndrome.^29^, ^30^, ^31^, ^32^ In contrast, we predicted a selective impairment of anticipatory anhedonia in AD, reflecting well‐documented cognitive impairments in the capacity to mentally construct future events.^32^, ^33^ Notably, consummatory pleasure was expected to remain relatively preserved in AD given its closer relationship to the affective and sensory experience of reward as it occurs.^22^, ^26^

## 2 METHODS

### 2.1 Participants

**RESEARCH IN CONTEXT**

1. **Systematic review**: We reviewed prior literature on anhedonia in dementia, focusing on studies including bvFTD and AD. Existing work largely conflates anhedonia with apathy. No studies examined the subcomponents of anhedonia or its temporal emergence relative to canonical symptom profiles.
2. **Interpretation**: This study extends prior work by demonstrating distinct profiles of anhedonia and their divergent temporal ordering in bvFTD and AD. Consummatory anhedonia emerged as a key feature distinguishing bvFTD from AD, while apathy was comparable across groups. Event‐based modeling revealed that global anhedonia appeared early in the bvFTD disease course but arose later in AD. These findings identify anhedonia as an early specific behavioral marker of bvFTD that could be readily incorporated into clinical assessment.
3. **Future directions**: Longitudinal studies examining the evolution of anhedonia components will clarify their prognostic value and inform the development of targeted interventions to support engagement and wellbeing in dementia.

A total of 127 participants were recruited through FRONTIER, the frontotemporal dementia research clinic based at the Brain and Mind Centre at the University of Sydney, Australia. Within this group, 59 participants met current diagnostic criteria for probable bvFTD,^5^ and 27 participants met the criteria for typical amnestic AD.^34^ Patients with atypical AD diagnoses (i.e., logopenic progressive aphasia, posterior cortical atrophy) were not included. Participant diagnoses were established by consensus among a multidisciplinary team, including a senior neurologist, neuropsychologist, and speech pathologist, based on comprehensive clinical investigation, neuropsychological assessment, informant interview, and review of brain atrophy on structural MRI. Disease duration was calculated as number of years elapsed from reported onset of symptoms to current date of testing.

Briefly, patients diagnosed with bvFTD presented with changes in personality and behavior, emotional blunting, and impulsivity, along with a decline in attention and cognitive functioning in the context of frontal and temporal gray matter atrophy.^5^, ^35^ In contrast, AD cases presented with episodic memory disturbances and executive and visuospatial dysfunction in the context of widespread cortical atrophy, including medial temporal, parietal, and prefrontal regions.^34^

Forty‐one cognitively healthy older control participants (age > 55 years) were recruited from the FRONTIER volunteer database and local community groups. All controls were required to score 88/100 or above on the Addenbrooke's Cognitive Examination III screening tool (ACE‐III),^36^, ^37^ zero on the Clinical Dementia Rating Scale,^38^ and within normal limits on all behavioral and cognitive measures. Exclusion criteria for all participants included a prior history of mental illness, alcohol or other drug abuse, significant head injury, or limited English language proficiency. Dementia patients scoring < 40/100 on the ACE‐III were excluded due to the severity of their cognitive impairment.

### 2.2 Clinical and cognitive assessment

Cognitive function was assessed using the ACE‐III, a validated clinical tool covering domains of attention and orientation, memory, fluency, language, and visuospatial function, where higher scores denote better performance (maximum score: 100).^36^ Functional impairment was rated by carers using the Frontotemporal Dementia Functional Rating Scale, where lower scores denote greater functional impairment.^39^ The depression subscale of the Depression, Anxiety and Stress Scale (DASS‐21) was used to assess self‐reported depressive symptoms.^40^ The Dimensional Apathy Scale (DApS) was included as a validated carer‐rated assessment of apathy (i) before dementia onset and (ii) at the current time, where higher scores reflect greater apathy.^41^

### 2.3 Assessment of anhedonia

The TEPS was used to evaluate anhedonia across anticipatory and consummatory dimensions.^23^ The Anticipatory subscale (TEPS‐A) contains 10 items that evaluate an individual's ability to look forward to rewards, for example: “*I get so excited the night before a major holiday I can hardly sleep*.” The Consummatory subscale (TEPS‐C) includes eight items designed to measure the capacity to experience in‐the‐moment pleasure, for example: “*I really enjoy the feeling of a good yawn*.” Participants indicate their level of agreement with each item on a four‐point Likert scale, ranging from 1 (very false for me) to 4 (very true for me), with lower scores reflecting greater loss of hedonic tone. As such, the TEPS‐A is scored from 10 to 40, and the TEPS‐C from 8 to 32. Given the imbalance between items on each TEPS subscale (TEPS‐A = anticipatory, *n* = 10 items; TEPS‐C = consummatory, *n* = 8 items), subscale totals were converted to percentages to enable direct comparison. Given the well‐documented changes in self‐awareness in many FTD disorders,^42^ carers rated the behaviors of patients while controls provided self‐ratings. This approach was informed by previous work demonstrating marked divergence between patient and carer report in relation to anhedonia in bvFTD, consistent with reduced insight in this syndrome.^13^

### 2.4 Statistical analyses

Statistical analyses were performed using IBM SPSS Statistics version 28.0. First, normality of distributions was checked using Shapiro‐Wilk tests and visual inspection of box plots. Where variables were normally distributed, separate univariate ANOVAs and independent *t*‐tests were used to examine group differences on continuous demographic variables (e.g., age, education) with Sidak post hoc tests conducted to explore the main effects of group (control, AD, bvFTD). Chi‐squared tests were used to investigate group differences on categorical variables (e.g., sex).

Given the TEPS data were not normally distributed, a log transformation was applied to the percentage scores prior to group comparisons. A 2 × 2 repeated‐measures ANCOVA, controlling for sex and education, was run to examine the main effects of the group (control, AD, bvFTD) and anhedonia domains (consummatory, anticipatory), as well as relevant interactions on the log‐transformed TEPS percentage scores, with Sidak post hoc tests. Partial eta‐squared values $\left(\eta_{p}^{2}\right)$ accompany all ANOVAs and ANCOVAs as measures of corresponding effect sizes. Spearman's correlations were run to examine the relationship between each dimension of anhedonia and symptoms of apathy (DApS) and depression (DASS‐D) within each group.

### 2.5 Event‐based modeling of anhedonia in dementia

Non‐parametric kernel‐density estimate event‐based models^43^ were run within each disease group relative to controls to examine the probabilistic ordering of anticipatory (TEPS‐A) and consummatory anhedonia (TEPS‐C) dimensions relative to other characteristic changes in cognition (ACE‐III subscales) and apathy (DApS total).^41^ All measures were regressed for age and sex covariates, then converted to a standard scale where higher values denote greater impairment (in line with EBM assumptions). Event‐based models were created using the “kde_ebm” package (version 0.0.2) in Python (version 3.7.1) and validated using leave‐one‐out cross‐validation via Scikit‐learn (version 1.0.2).

## 3 RESULTS

### 3.1 Sample characteristics

Demographic, cognitive, and clinical characteristics of the study sample are presented in Table 1. Groups did not differ significantly in terms of age (*F*[2, 124] = 2.7, *p* = 0.07,  $\eta_{p}^{2}$= 0.04), but differed for sex distribution (*χ^2^* = 17.9, *p <* 0.001), with significantly more males in the bvFTD group and more females in the control group (both *p* < 0.05). A significant group effect emerged for years of education (*F*[2, 124] = 3.2, *p* = 0.04*,* $\eta_{p}^{2}$= 0.04), with controls reporting more years of education than bvFTDs (*p* = 0.04). A main group effect emerged on the ACE‐III screening tool (*F*[2, 123] = 44.5, *p <* 0.001,  $\eta_{p}^{2}$= 0.42), reflecting significant cognitive impairment in both patient groups compared to controls (all *p <* 0.001). Self‐reported depressive symptoms differed across groups (DASS‐21 Depression: *F*[2, 124] = 12.0, *p *< 0.001, $\eta_{p}^{2}$= 0.21), with AD and bvFTD patient groups reporting higher scores than controls (both *p* < 0.001). Importantly, however, mean scores indicated low levels of depressive symptoms, falling within the normal range for bvFTD and the mild range for AD. Direct comparison of the dementia groups revealed longer disease duration (*t*[75] = −2.8, *p* = 0.006, 95% CI: 0.8 to 4.7), elevated apathy (*t*[84) = −2.7, *p* = 0.008, 95% CI 2.0 to 13), and greater functional impairment (FRS: *t*[80] = 2.5, *p* = 0.02, 95% CI: 0.8 to 4.7) in bvFTD relative to AD, while AD patients showed disproportionate cognitive impairment relative to bvFTD (ACE‐III total: *p* = 0.009).

**TABLE 1:** Demographic, clinical, and cognitive characteristics of the study samples.

Variable	bvFTD (*n *= 59)	AD (*n *= 27)	Control (*n *= 41)	Test statistic	Post hoc comparisons
Age, years	65.1 (8.3)	65.4 (7.7)	68.4 (6.1)	*F* = 2.6	n/s
Education, years	12.7 (3.2)	13.5 (2.6)	14.3 (3.2)	*F* = 3.2^*^	Control > bvFTD
Sex, M:F	46:13	15:12	15:26	χ = 17.9^***^	M > F in bvFTD
Disease duration, years	7.7 (4.6)	5.0 (2.7)	—	*t* = −2.8^**^	bvFTD > AD
ACE‐III total [100]	77.1 (15.6)	67.4 (14.9)	95.6 (3.2)	*F* = 44.0^***^	Control > bvFTD > AD
ACE‐III attention [18]	14.7 (3.2)	12.1 (3.8)	17.4 (0.9)	*F* = 29.3^***^	Control > bvFTD > AD
ACE‐III memory [26]	18.2 (6.4)	12.19 (5.7)	25.1 (1.3)	*F* = 48.3^***^	Control > bvFTD > AD
ACE‐III fluency [14]	7.5 (4.2)	7.7 (3.3)	12 (1.8)	*F* = 23.8^***^	Control > AD, bvFTD
ACE‐III language [26]	22.9 (3.2)	22.5 (2.9)	25.4 (0.8)	*F* = 14.5^***^	Control > bvFTD, AD
ACE‐III visuospatial [16]	14.1 (2.2)	12.6 (3.9)	15.6 (0.7)	*F* = 13.6^***^	Control > bvFTD > AD
FRS logit score^a^	−0.18 (1.8)	0.81 (1.6)	—	*t* = 2.5^*^	AD > bvFTD
DASS‐ D‐21 [21]	4.5 (4.7)	5.3 (4.7)	1.0 (1.1)	*F* = 12.0^***^	AD, bvFTD > Control
DASS‐D category	Normal	Mild	Normal		
DApS [72]	50.5 (12.0)	43 (11.7)	—	*t* = −2.7^*^	bvFTD > AD

*Note*: Values are presented as mean (standard deviation) unless otherwise specified. Maximum score for each test shown in square brackets where appropriate. Missing data: FRS not available for two bvFTD patients and one AD patient. Disease duration not available for one bvFTD patient.

Abbreviations: —, not applicable; ACE‐III, Addenbrooke's Cognitive Examination third edition; AD, Alzheimer's disease; bvFTD, behavioral variant frontotemporal dementia; D, Depression Subscale; DApS, Dimensional Apathy Scale; DASS, Depression, Anxiety and Stress Scale; F, female; FRS, Frontotemporal Dementia Functional Rating Scale; M, Male; n/s, not significant.

^*^ *p <* *0*.05.^**^ *p *< 0.005.^***^ *p *< 0.001.

^a^Lower scores denote greater levels of functional impairment on FRS.

### 3.2 Profiles of anhedonia across dementia syndromes

Figure 1 displays profiles of anhedonia across dementia syndromes using the converted percentage scores from the TEPS (raw scores presented in Supplementary Material). A repeated‐measures ANCOVA, controlling for education and sex, revealed a significant main effect of group (*F*[2, 110] = 20.9, *p *< 0.001, $\eta_{p}^{2}$= 0.28), reflecting heightened anhedonia, irrespective of domain, in bvFTD relative to AD and controls (both *p* < 0.001). In contrast, AD patients did not differ in terms of overall levels of anhedonia from controls (*p* = 0.07). No main effect of domain was observed (*F*[1, 110] = 0.01, *p* = 0.92, $\eta_{p}^{2}$= 0.00); however, the group *×* domain interaction was significant (*F*[1, 110] = 3.8, *p* = 0.03, $\eta_{p}^{2}$= 0.08), indicating distinct anhedonia profiles across syndromes.

**FIGURE 1:**
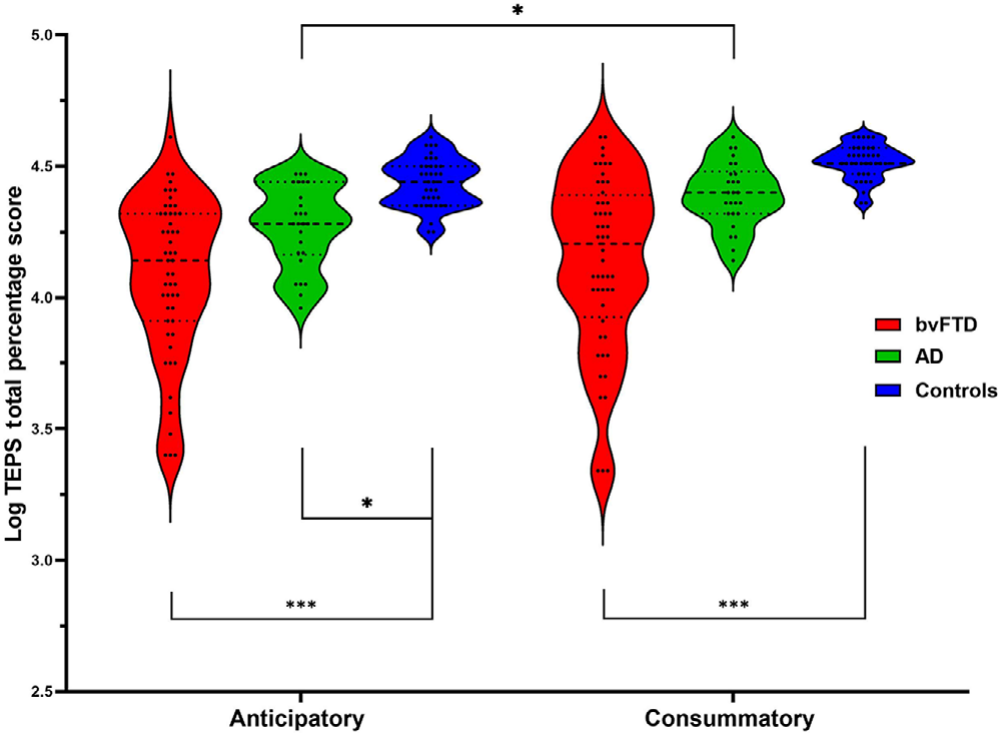
Profiles of anticipatory and consummatory anhedonia. Violin plots depict distribution of data, with width of shaded area representing proportion of data located there. Bolded horizontal line depicts the median score. Thin horizontal lines depict the quartiles. TEPS scores are log‐transformed percentage scores, where lower values reflect greater anhedonia severity. TEPS scores are carer‐rated for patients and self‐rated for controls. AD *n = *27; bvFTD *n = *59. Asterisks denote results that emerged as significant in the analyses with sex and education included as covariates. AD, Alzheimer's disease; bvFTD, behavioral variant frontotemporal dementia; TEPS, Temporal Experience of Pleasure Scale. *p < 0.05; ***p < 0.001.

In bvFTD, both anticipatory (TEPS‐A: *p *< 0.001, mean difference = 0.31, 95% CI: 0.2 to 0.4) and consummatory (TEPS‐C: *p *< 0.001, mean difference = 0.33, 95% CI: 0.2 to 0.5) anhedonia were significantly elevated relative to controls. Within‐group comparisons showed comparable levels of anticipatory and consummatory anhedonia (*p* = 0.12), consistent with a global reduction in pleasure.

In contrast, AD patients displayed elevated anticipatory anhedonia (TEPS‐A: *p* = 0.04, mean difference = 0.14, 95% CI: 0.1 to 0.3) in the context of relatively preserved consummatory pleasure (TEPS‐C: *p* = 0.30) compared to controls. Within the AD group, anticipatory anhedonia was significantly greater than consummatory anhedonia (*p* = 0.04, mean difference = 0.12, 95% CI: 0.04 to 0.2), indicating a relative preservation of in‐the‐moment pleasure.

Finally, irrespective of domain, bvFTD patients showed greater severity of anticipatory (TEPS‐A; *p* = 0.009, mean difference = 0.17, 95% CI: 0.03 to 0.30) and consummatory (TEPS‐C; *p *< 0.001, mean difference = 0.23, 95% CI: 0.1 to 0.4) anhedonia compared to the AD group.

To explore the potential influence of disease severity on findings, separate sensitivity analyses were conducted adjusting for (1) disease duration and (2) functional impairment on the FRS. Importantly, participants with bvFTD continued to display significantly greater consummatory anhedonia than AD after adjusting for disease duration (*F*[1, 65] = 6.23, *p* = 0.015, $\eta_{p}^{2}$ = 0.087, mean difference = 0.18, 95% CI: 0.04 to 0.33) and functional impairment on the FRS (*F*[1, 70] = 7.45, *p* = 0.008, $\eta_{p}^{2}$ = 0.096, mean difference = 0.21, 95% CI: 0.06 to 0.36). These analyses indicate that the consummatory anhedonia profile in bvFTD cannot be explained by greater disease severity. See Supplementary Material for full reporting of sensitivity analyses.

### 3.3 Associations between anhedonia and clinical measures of interest

Table 2 displays associations between anticipatory and consummatory anhedonia and total apathy on the DApS and depressive symptomatology on the DASS‐D. In the bvFTD group, higher anticipatory anhedonia was associated with greater apathy severity (*r* = −0.31, *p* = 0.02). No other significant associations were found (all *p* > 0.13) In AD, higher anticipatory (*r* = −0.61, *p* = 0.001) and consummatory (*r* = −0.59, *p* = 0.002) anhedonia were associated with increased apathy (DApS total score). Importantly, depressive symptoms on the DASS‐21 were not found to correlate with either anticipatory or consummatory anhedonia in any of the participant groups (all *p* > 0.32).

**TABLE 2:** Associations between dimensions of anhedonia on the TEPS and independent measures of apathy and depression in each participant group.

Dimension of pleasure	Clinical measure	bvFTD	AD	Control
Anticipatory (TEPS‐A)	DApS	***r* = −0.31**	***r* = −0.61**	*r* = 0.14
	DASS‐D	*r* = −0.08	*r* = −0.19	*r* = 0.05
Consummatory (TEPS‐C)	DApS	*r *= −0.22	***r* = −0.59**	*r* = −0.38
	DASS‐D	*r* = −0.05	*r* = −0.18	*r* = −0.18

*Note*: Higher DApS and DASS scores indicate greater apathy and depression, respectively, whereas lower TEPS scores indicate greater anhedonia. Correlations are Spearman's rank‐order coefficients. Bolded values remained significant following Benjamini–Hochberg correction for false discovery rate at *q* = 0.05.

Abbreviations: A, Anticipatory Subscale; AD, Alzheimer's disease; bvFTD, behavioral variant frontotemporal dementia; C, Consummatory Subscale; DApS, Dimensional Apathy Scale Total; DASS‐D, Depression Subscale of the Depression Anxiety and Stress Score 21‐Item Version; TEPS, Temporal Experience of Pleasure Scale.

### 3.4 EBM symptom orderings in each dementia syndrome

Figure 2 displays the EBM results for AD (green) and bvFTD (red), including cross‐validation runs. Irrespective of group, apathy emerged as the earliest clinical feature; however, subsequent features differed depending on dementia subtype. In bvFTD, apathy, consummatory anhedonia, and anticipatory anhedonia were probabilistically most likely to emerge as the first three symptoms, followed by cognitive changes in memory, attention, language, visuospatial ability, and fluency.

**FIGURE 2:**
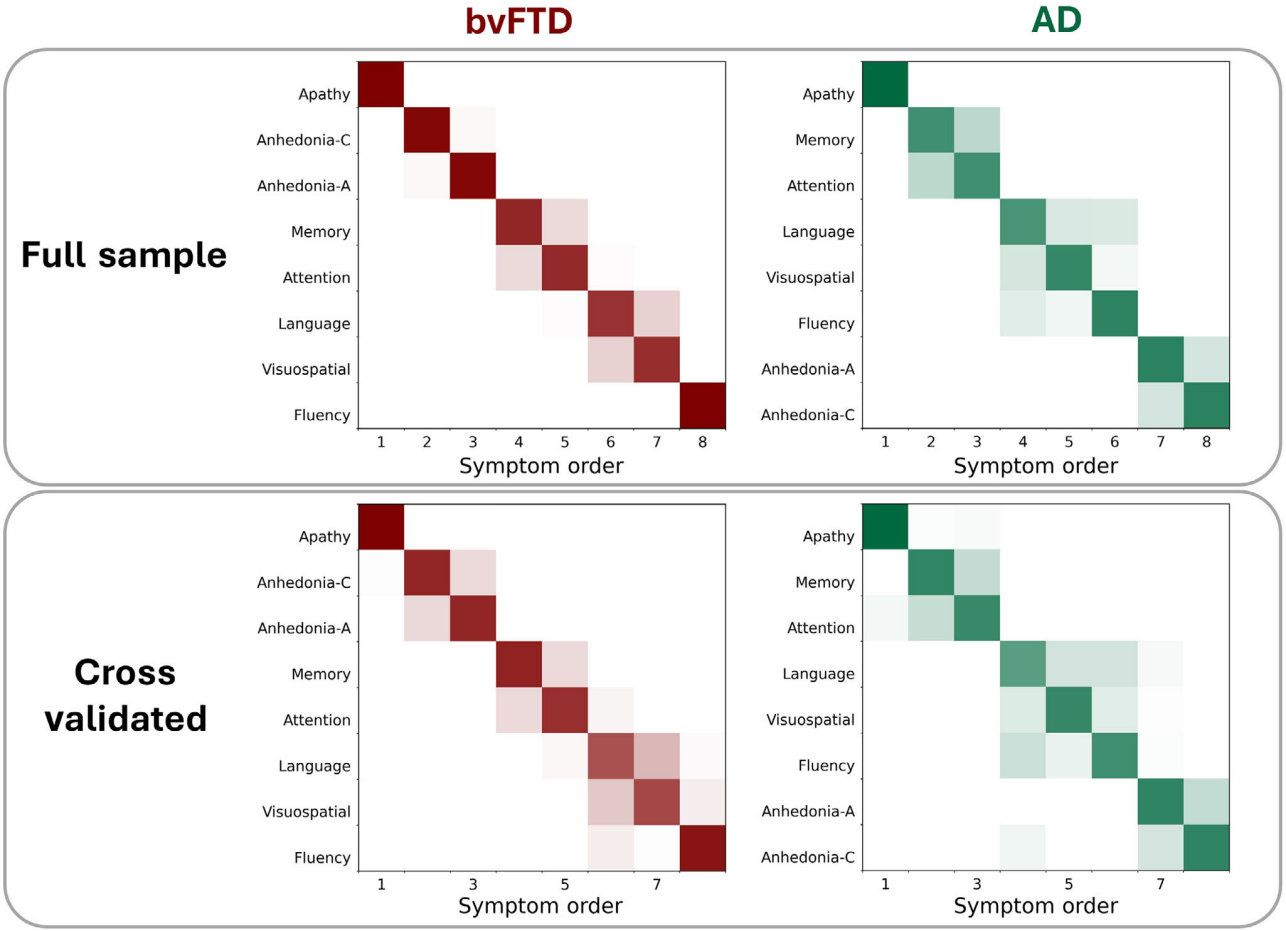
Probabilistic staging of apathy, anticipatory (A), and consummatory (C) anhedonia and cognitive symptoms in AD and behavioral variant frontotemporal dementia (bvFTD). Anhedonia was assessed using the Temporal Experience of Pleasure Scale. Positional variance plots indicate the likelihood and order that features become abnormal in each dementia group. Likelihood is denoted by the occupancy of colors, where bolder color denotes a higher likelihood. Cross‐validated results are acquired using leave‐one‐out cross‐validation (LOOCV), an averaging of all model runs in an exhaustive all‐versus‐one design. The observed similarity between original and LOOCV runs indicates strong reliability. Green plots denote results for AD and red for bvFTD.

For AD, however, early presence of apathy preceded changes in cognition including memory, attention, language, visuospatial, and fluency, with anticipatory and consummatory anhedonia emerging as the latest clinical features in the AD sequence.

## 4 DISCUSSION

Early behavioral change is a hallmark of bvFTD and frequently represents the first point of concern for carers and clinicians. Commonly reported features include social withdrawal, loss of interest in previously meaningful activities, and reduced emotional responsiveness. Here, we demonstrate that these disturbances extend to multidimensional components of pleasure. Individuals with bvFTD showed marked anticipatory and consummatory anhedonia, whereas AD was characterized by selective anticipatory anhedonia with preserved consummatory pleasure. EBM further placed multidimensional anhedonia among the earliest features of bvFTD, but later in the AD disease sequence. Together, these findings highlight multidimensional anhedonia as an important early behavioral feature of bvFTD.

Using a multidimensional approach, our findings reveal prominent impairments in the ability to anticipate the pleasure associated with future rewards as well as the in‐the‐moment experience of pleasure in bvFTD. This profile is consistent with a global dampening of the capacity to both anticipate and experience reward.^12^, ^13^ Crucially, when these symptoms were situated within a broader disease sequence using EBM, consummatory and anticipatory anhedonia emerged probabilistically as two of the earliest abnormal features in bvFTD. Notably, these pleasure‐related disturbances were more likely to occur prior to overt cognitive decline, underscoring their relevance as early behavioral indicators of bvFTD. It has been suggested that subtle changes in hedonic tone might be an early harbinger of prodromal bvFTD.^44^ Our findings support this proposal and point to the need for targeted assessment tools that decouple anhedonia from apathy.

In contrast, AD was characterized by a more selective anhedonic profile, with relatively preserved consummatory pleasure despite diminished anticipatory pleasure. This dissociation suggests that while individuals with AD may have difficulty anticipating or looking forward to future rewards,^32^, ^33^ they nevertheless derive enjoyment from in‐the‐moment rewarding experiences. Importantly, our EBM findings suggest that anticipatory anhedonia is more likely to manifest after cognitive changes in AD but prior to the emergence of consummatory anhedonia. This pattern is consistent with our proposal that cognitive difficulties may contribute to reduced anticipation of future rewards in AD, while in‐the‐moment pleasure remains relatively preserved until later disease stages. By contrast, the emergence of anticipatory anhedonia prior to overt cognitive decline in bvFTD suggests that it cannot be attributed solely to broader cognitive impairment.

Across both syndromes, cognitive features followed broadly overlapping staging patterns, with abnormalities in memory, attention, language, visuospatial ability, and fluency emerging in comparable sequences. Together with the shared early emergence of apathy, these findings highlight the challenges of differentiating bvFTD and AD based on the initial presentation of cognitive and motivational abnormalities. By contrast, anhedonia dimensions showed divergent probabilistic staging patterns across syndromes. These findings suggest that multidimensional disturbances in pleasure, particularly consummatory anhedonia, may provide clinically informative behavioral signals, particularly when core neuropsychiatric and cognitive features overlap. Importantly, the greater severity of consummatory anhedonia observed in bvFTD could not be explained by differences in disease duration or functional impairment. This finding suggests that motivational disengagement should be prioritized as an early canonical feature of the bvFTD syndrome and that current diagnostic criteria should be broadened to include anhedonia as a hallmark symptom.^44^

Correlation analyses further support the distinction between anhedonia and broader neuropsychiatric symptoms. In bvFTD, greater apathy was associated with anticipatory, but not consummatory, anhedonia, while in AD, apathy correlated with both anhedonia domains. Importantly, depressive symptoms were not associated with either anticipatory or consummatory anhedonia in any group. These patterns suggest that consummatory anhedonia is a standalone clinical feature of bvFTD that cannot be subsumed within the broader category of apathy or attributed to depression. In contrast, for AD, motivational and hedonic changes appear to be more closely coupled, potentially reflecting symptom overlap with more advanced stages of neurodegeneration.

The clinical implications of our findings are two‐fold. First, from the perspective of diagnosis, multidimensional anhedonia represents an early behavioral feature of bvFTD, with reduced in‐the‐moment pleasure providing a potentially informative marker of disease. In contrast, the late emergence of anhedonia in AD suggests that loss of immediate pleasure may signal disease progression rather than serve as an early diagnostic feature. Incorporating multidimensional anhedonia measures into routine clinical assessments may improve early diagnostic confidence and guide personalized behavioral interventions.^45^ Second, these findings have important implications for carer psychoeducation. In AD, difficulty envisaging future activities or events should not be interpreted as a lack of interest or enjoyment. Rather, individuals may retain the capacity to experience pleasure when rewards are immediately available. This distinction may help guide caregiving approaches and supports the continued use of interventions that engage preserved in‐the‐moment pleasure, such as music and art‐based activities,^46^, ^47^ even in the presence of reduced anticipatory motivation.

Several methodological issues warrant consideration. Anhedonia was rated by carers for participants with dementia but self‐rated by controls. Although this approach reduces the potential influence of impaired insight in bvFTD, the use of different rating sources may introduce systematic measurement effects unrelated to disease status. Carer ratings may also be influenced by informant perceptions of observable behaviors, as well as ongoing stress or burden related to the caregiving experience. While our findings are in keeping with the broader literature on motivational impairments in dementia, we acknowledge that the use of carer report may not capture the heterogeneity of reward processing across sensory, social, and abstract domains. Reward‐processing disturbances in bvFTD are not uniform across all reward types or stages of processing, with both reduced and heightened reward seeking evident across sensory, social, and abstract domains.^48^, ^49^ Future studies incorporating behavioral and physiological measurement approaches (e.g., digital sensors, extended experience sampling),^50^ alongside personally salient and individualized rewards, will be important for determining the extent to which these hedonic profiles generalize across reward contexts in bvFTD. Kernel‐density estimate event‐based models are not longitudinal models, meaning the probabilistic orderings observed in this study are relative to the baseline control population and the clinical measures used. As with any model, event‐based ones are influenced by feature choices, number of variables, and sample size. Accordingly, the orderings reported here will need to be validated in independent datasets and using longitudinal approaches. Finally, recruitment through a specialist tertiary referral center may limit the generalizability of the findings to broader clinical and community‐based populations. Future work should replicate these findings in larger, multisite cohorts that include more diverse referral pathways, clinical presentations, and disease stages.

In conclusion, this is the first study to use EBM to characterize the probabilistic emergence of anticipatory and consummatory anhedonia in bvFTD and AD using cross‐sectional data. Overall, our findings point to early multidimensional hedonic disturbance in bvFTD and highlight consummatory anhedonia as a potentially informative feature for differentiating bvFTD from AD in the context of overlapping cognitive and neuropsychiatric features.

## AUTHOR CONTRIBUTIONS

*Study design*: Siobhán Shaw and Muireann Irish. *Data acquisition*: Siobhán Shaw, Claris Teng, Jody Huynh, Olivier Piguet, and Rebekah Ahmed. *Statistical analysis*: Siobhán Shaw, Samuel L Warren, Alexis E Whitton, and Muireann Irish. *Interpretation*: Siobhán Shaw, Samuel L Warren, Alexis E Whitton, and Muireann Irish. *Drafting of manuscript*: Siobhán Shaw and Muireann Irish. *Editing of manuscript*: Siobhán Shaw, Samuel L Warren, Olivier Piguet, Rebekah Ahmed, Alexis E Whitton, and Muireann Irish *Study supervision*: Muireann Irish and Alexis E Whitton. All authors have read and agreed to the published version of the manuscript.

## CONFLICT OF INTEREST STATEMENT

The authors declare no conflicts of interest.

Author disclosures are available in the supporting information.

## ETHICS STATEMENT

Ethics approval for this study was provided by the University of New South Wales, the South Eastern Sydney Local Health District and the University of Sydney Ethics Committees.

## CONSENT STATEMENT

All participants, or their person responsible, provided informed consent in accordance with the Declaration of Helsinki.

## Supporting information



Supporting Information

Supporting Information
