# Supplementary material for: Early anhedonia distinguishes behavioral variant frontotemporal dementia from Alzheimer's disease
Source: Alzheimers Dement (Amst). 2026 Oct 9;18(4):e70501. doi: 10.1002/dad2.70501 (PMC13650516; doi:10.1002/dad2.70501)
Supplement: Supplementary file 2 [file DAD2-18-e70501-s001.docx]

**Early anhedonia distinguishes behavioural variant frontotemporal dementia from Alzheimer’s disease**

Siobhán Shaw^1,2*^, Samuel L Warren^1,2^, Claris Teng ^1,2^, Jody Huynh^1,2^, Rebekah Ahmed^1,3^, Olivier Piguet^1,2^, Alexis E Whitton^4^, Muireann Irish^1,2*^

1. The University of Sydney, Brain and Mind Centre, Sydney, New South Wales, Australia
2. The University of Sydney, School of Psychology, Sydney, New South Wales, Australia
3. The University of Sydney, School of Medical Sciences, Sydney, New South Wales, Australia
4. Black Dog Institute, University of New South Wales, Sydney, New South Wales, Australia

*Correspondence:	Dr Siobhán Shaw

Brain and Mind Centre, The University of Sydney
94 Mallett Street
Camperdown
NSW 2050 Australia

[Siobhan.shaw@sydney.edu.au](mailto:Siobhan.shaw@sydney.edu.au)

and

Professor Muireann Irish

Brain and Mind Centre, The University of Sydney
94 Mallett Street
Camperdown
NSW 2050 Australia

[muireann.irish@sydney.edu.au](mailto:muireann.irish@sydney.edu.au) ; tel.: +61-2-9114-4165

**Supplementary Material**

**Temporal Experience of Pleasure Scale – Raw Scores**

Raw anticipatory and consummatory pleasure scores differed significantly across groups [TEPS-A: *F*(2,124)=32.6, *p*<.001,$\eta_{p}^{2}$=.37; TEPS-C: *F*(2,124)=36.3, *p*<.001, $\eta_{p}^{2}$=.39]. A graded pattern was evident across both domains, with Controls showing the lowest levels of anhedonia, followed by AD and then bvFTD (TEPS-C: all *p* values<.02; TEPS-A: all *p* values<.004).

Supplementary Table 1. Temporal Experience of Pleasure Scale raw scores across the study samples

| **Pleasure dimension** | **bvFTD**  **(n=59)** | **AD**  **(n=27)** | **Controls**  **(n=41)** |
| --- | --- | --- | --- |
| Anticipatory [TEPS-A]  Maximum score: 40 | 24.7 (6.8) | 29 (4.4) | 34 (3.1) |
| Consummatory [TEPS-C]  Maximum score: 32 | 21.0 (6.3) | 26 (3.1) | 29 (1.9) |

*Note.* Values are presented as mean with standard deviation in parentheses. AD = Alzheimer’s disease; bvFTD = behavioural-variant frontotemporal dementia; TEPS-A = Anticipatory subscale of the Temporal Experience of Pleasure Scale, TEPS-C = Consummatory subscale of the Temporal Experience of Pleasure Scale.

**Controlling for disease severity**

To examine whether differences in disease severity contributed to the observed group differences in anhedonia, we re-ran the original univariate ANCOVAs controlling for sex and education, to compare the bvFTD and AD groups across each TEPS domain (i.e., Anticipatory, Consummatory), while also controlling for (i) disease duration and (ii) functional impairment on the FRS.

Participants with bvFTD continued to display significantly greater consummatory anhedonia than AD after adjusting for disease duration [*F*(1, 65) = 6.23, *p* = .015, $\eta_{p}^{2}$ = .087, Mean Difference=.18, 95%CI .04-.33] and functional impairment, [*F*(1, 70) = 7.45, *p* = .008, $\eta_{p}^{2}$ = .096, Mean Difference=.21, 95%CI .06-.36]. The group difference in anticipatory anhedonia remained significant after adjustment for functional impairment, with bvFTD showing greater anticipatory anhedonia than AD [*F*(1, 70) = 4.33, *p* = .04, $\eta_{p}^{2}$ = .033, Mean Difference=.11, 95%CI .01-.28]. In contrast, when controlling for disease duration, anticipatory anhedonia no longer differed significantly between bvFTD and AD [*F*(1, 65) = 2.25, *p* = .138, $\eta_{p}^{2}$ = .033, Mean Difference=.11, 95%CI .04-.25].
